# The impact of higher education investment on the urban-rural income gap: An analysis of mediating and threshold effects based on data from China’s eight major comprehensive economic zones

**DOI:** 10.1371/journal.pone.0326059

**Published:** 2025-06-25

**Authors:** Shuyao Liu, Yan Zhang, Yanbo Liu, Ning Wang

**Affiliations:** 1 The College of Economics and Management, Shenyang Agricultural University, Shenyang, China; 2 Institute of Higher Education Research, Shenyang Agricultural University, Shenyang, China; 3 Center for Higher Education Research, Hainan Tropical Ocean University, Sanya, China; Yunnan University, CHINA

## Abstract

**Background:**

Since the reform and opening up, China’s urban and rural economic development has exhibited characteristics of imbalance, with the urban-rural income gap being the largest and most noticeable issue facing China’s socio-economic landscape. Alleviating and effectively resolving the urban-rural income disparity is crucial for achieving overall common prosperity. Therefore, this study provides insights for strategically narrowing the urban-rural income gap from the perspective of higher education investment.

**Methods:**

We employ a panel fixed effects model to examine the basic regression, heterogeneity, mediating effects, and threshold effects. Simultaneously, we address the endogeneity issues in basic regression and mediating effects using the instrumental variable method. Additionally, we adopt the substitution of variables to ensure the robustness of the results.

**Results:**

This paper selects panel data from China’s eight major comprehensive economic zones from 2003 to 2021 for analysis. The findings reveal that, overall, higher education investment in China’s eight major comprehensive economic zones can narrow the urban-rural income gap. Specifically, higher education investment in 50% of these comprehensive economic zones—namely, the Northern Coastal Comprehensive Economic Zone, Eastern Coastal Comprehensive Economic Zone, Northeast Comprehensive Economic Zone, and Middle Yangtze River Comprehensive Economic Zone—can reduce the urban-rural income disparity. Conversely, higher education investment in the Middle Yellow River Comprehensive Economic Zone, Southern Coastal Comprehensive Economic Zone, Greater Southwest Comprehensive Economic Zone, and Greater Northwest Comprehensive Economic Zone has widened the urban-rural income gap. Additionally, higher education investment can affect the urban-rural income disparity through technological innovation. Overall, the impact of higher education investment on the urban-rural income gap in China’s eight major comprehensive economic zones is also influenced by the level of economic development, exhibiting an “inverted U-shaped” characteristic. This nonlinear impact varies across regions.

**Conclusions:**

In conclusion, to narrow the urban-rural income gap across China’s eight major integrated economic zones, it is necessary to improve the mechanism for higher education investment in these zones. Strategies should be based on regional differences, tailored to local conditions, and implemented with a differentiated and precise approach to higher education development across regions. Emphasis should also be placed on the research and application of innovative technologies to achieve deep integration between urban and rural areas within China’s eight major integrated economic zones.

## Introduction

**Fig 1:**
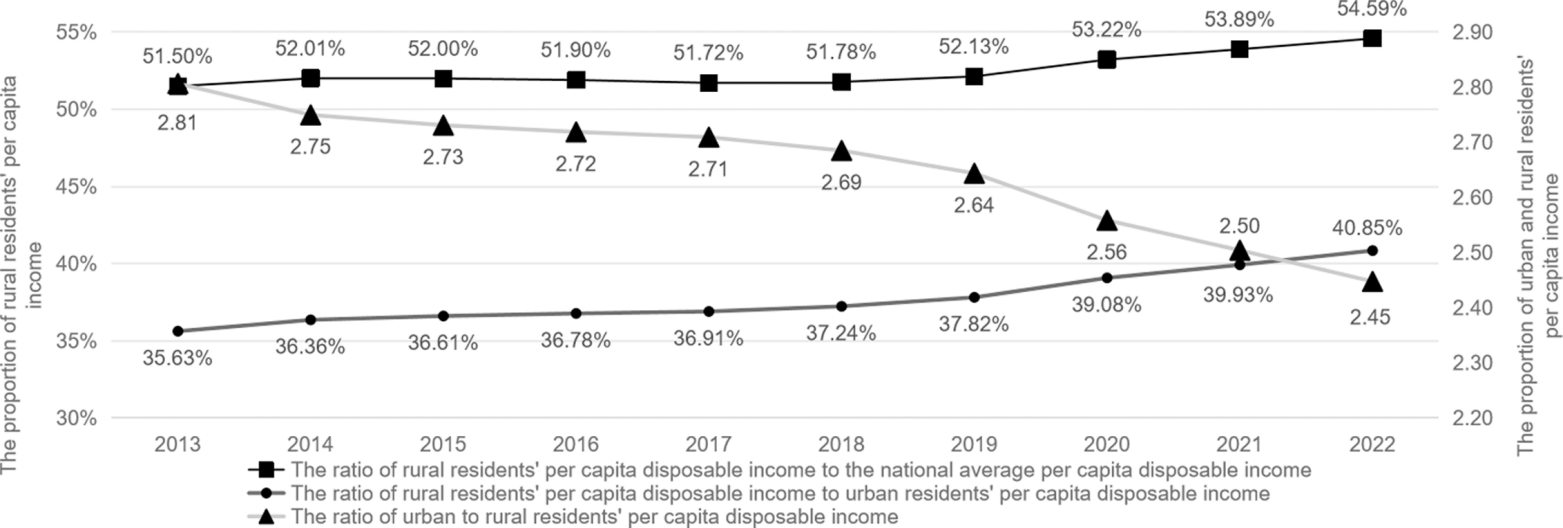
The urban-rural per capita disposable income gap in China, 2013–2022.

Since the implementation of the reform and opening-up policy, guided by the strategy of unbalanced development, China has enacted a series of urban-biased development policies. After years of persistent efforts, China’s economic strength, scientific and technological capabilities, and comprehensive national power have significantly increased, entering a new era dedicated to promoting common prosperity among all its citizens [1,2]. To promote common prosperity for all people, General Secretary Xi Jinping has repeatedly emphasized the need to proactively address issues such as regional disparities, urban-rural disparities, and income disparities. Compared with disparities in group income and regional income, the impact of urban-rural income disparities is the most severe, the urgency to improve the current situation is the greatest, and the benefits of such improvement are the most profound [3].Regarding the issue of urban-rural income disparity, since the 18th National Congress of the Communist Party of China, the state’s investment in “agriculture, rural areas, and farmers” has been unprecedented, resulting in a significant increase in farmers’ incomes. According to data from the China Statistical Yearbook, the gap between urban and rural incomes has gradually narrowed over the past decade, with the absolute difference in per capita disposable income of urban and rural residents decreasing from 2.81:1 in 2013 to 2.45:1 in 2022 (Fig 1).From an international perspective, the urban-rural income disparity ratio in developed countries such as Canada and the UK, among other Western countries, approaches 1.0. In contrast, in developing countries, with India as an example, this urban-rural income differential ratio is close to 1.9. Furthermore, in some low-income African countries, such as Kenya, this disparity ratio is approximately 2.3. This indicates that, compared to most countries, China’s urban-rural income gap remains at a relatively high level.Therefore, narrowing the urban-rural income gap has always been a focal issue of high social concern. Among various factors affecting income distribution, education plays a crucial role, especially higher education, which is key to enhancing individual income. Higher education contributes to narrowing the urban-rural income gap by enhancing the quality of the workforce, increasing the accumulation of human capital in society, and promoting technological innovation.

In fact, there is no consensus on the relationship between higher education investment and the urban-rural income gap. Some scholars believe that investment in higher education helps to narrow this gap. For instance, Ma Lei (2016), utilizing panel data from 30 provinces in China for the years 2002–2013, discovered from a human capital optimization perspective that enhancing the relative proportion of the labor force with college education or above in both urban and rural settings significantly lowers the wage income ratio between urban and rural areas, with this effect being more pronounced in the central and western regions of China [4]. Shi Daqian and Zhang Weidong (2017), employing provincial panel data spanning from 1991 to 2015 and using a difference-in-differences approach, empirically examined the scale effect of university expansion. They further demonstrated that by increasing educational opportunities and non-agricultural employment, it is possible to diminish the urban-rural income gap [5].Zhang Hui and Yi Tian (2017), leveraging panel data from 31 provinces, autonomous regions, and municipalities in China spanning from 2004 to 2013, and adopting a perspective of stratified education, found that vocational and general higher education have facilitated the narrowing of the urban-rural income gap in China, and this facilitative effect is more significant in vocational education [6]. Li Xin and Guan Huijuan (2018), utilizing provincial panel data from 1995 to 2014, analyzed the micro-mechanism of educational investment in reducing the income gap between urban and rural residents through general equilibrium theory, and empirically verified that investment in all levels of education can narrow the income gap [7]. Wang Wenjing (2019), utilizing data from China’s Household Income Survey of 2007, found that the expansion of university enrollment policies has narrowed the income gap between high-income groups in urban and rural areas to some extent [8]. Chen Feng (2020), based on national-level panel data from 2013 to 2018, discovered that the percentage difference in higher education enrollment between urban and rural areas positively affects the income gap, suggesting that by leveraging the important role of universities in educational poverty alleviation, the urban-rural income gap can be narrowed [9]. Hu Dexin and Tian Yunhong (2022), based on data from the Chinese General Social Survey (CGSS) for the years 2008 and 2017, found that the expansion of higher education has a “fairness effect” that narrows the urban-rural income gap [10].Ionela-Roxana and Petcu (2022), utilizing relevant data from Romania, found that higher levels of education are more capable of reducing the urban-rural wage gap [11].

In contrast, some scholars have questioned the inhibitory effect of higher education investment on the urban-rural income gap. For example, Wang Haiyun et al. (2009), based on panel data from Chongqing for the period 1985–2006, pointed out that both in the short and long term, the development of higher education has shown to exacerbate the urban-rural income disparity. In other words, the development of higher education is not conducive to narrowing the gap in income between urban and rural areas [12]. Zhang Xingxiang (2012), utilizing cross-sectional data from the China Household Income Project (CHIP) for the year 2002 and based on the Mincer equation, found that higher education expansion has widened the urban-rural income gap, leading to the formation of a “Matthew effect” in higher education-income between urban and rural areas [13].

Additionally, Some scholars have also found that the impact of higher education on the urban-rural income gap exhibits non-linear characteristics. Liu Minlou (2008), using inter-provincial data from China for the years 1990 and 2000, observed that the relationship between human capital accumulation as well as investment and the urban-rural income gap, resulting from higher education investment, exhibits a nonlinear characteristic. Specifically, there is a “U-shaped” relationship, indicating an initial decline followed by an increase. This means that in the initial stages, human capital accumulation and investment can significantly narrow the urban-rural income gap. However, after declining to a certain level and crossing a threshold value, they begin to gradually widen the income gap [14]. Arshed et al. (2018), utilizing data from the South Asian Association for Regional Cooperation (SAARC) countries from 1990 to 2015, found that the rate of return on higher education exhibits a “U-shaped” relationship. Initially, higher enrollment in education reduces inequality, but after reaching a certain threshold of enrollment rate, the effect reverses [15].Cai Wenbo and Huang Jinsheng (2019) found that at the national level, higher education investment has widened the urban-rural income gap. Moreover, a nonlinear relationship between the two exists in regions with different economic growth rates, with a more pronounced effect in regions with medium economic growth [16]. Hu Yongmei and Xue Yuankang (2022), based on provincial panel data from 31 provinces in Mainland China from 2003 to 2019, discovered an “inverted U-shaped” spatial spillover effect of the scale of higher education on the urban-rural income gap. In other words, beyond a certain scale, the expansion of university enrollment can function to narrow the urban-rural income gap, further promoting social equity [17].

In summary, existing research has conducted in-depth studies on the relationship between higher education investment and the urban-rural income gap, revealing that higher education investment is an important factor affecting this gap. However, the relationship varies depending on the time period, context, geographical conditions, and economic environment. Pertaining to the research subject, the current literature predominantly originates from national or provincial perspectives, with a paucity of studies at the regional level. In terms of research angles, the majority of inquiries justify their arguments from the perspective of university expansion policies, overlooking the diversified characteristics of higher education investment. Furthermore, there has been no literature exploring the impact of higher education investment on the urban-rural income gap from the perspective of technological innovation.

In light of this, the present study seeks to contribute marginally in three aspects, based on the existing literature. First, it endeavors to construct a novel indicator system for higher education investment from three dimensions: human, material, and financial resources, providing an effective evaluation method for scientifically measuring higher education investment. Second, adopting a technological innovation perspective and using the level of economic development as a threshold variable, it employs panel models to examine the pathways and nonlinear relationships through which higher education investment affects the urban-rural income gap. This approach aims to unveil the mechanisms by which higher education investment influences the urban-rural income disparity, offering valuable insights for maximizing the positive impact of higher education investment in bridging this divide. Third, in accordance with the standards of the Development Research Center of the State Council, this study abandons the traditional geographical division of east, middle, and west, and follows the general developmental patterns of regions, focusing on China’s eight comprehensive economic zones. For the first time, it explores the heterogeneous impacts of higher education investment on the urban-rural income disparity across these zones, further broadening the discourse on the influence of higher education investment on the urban-rural income gap. This provides a theoretical foundation and policy implications for optimizing higher education investment in China, scientifically allocating higher education resources, and narrowing the urban-rural income disparity.

## Research design

### Model construction

This research aims to explore the relationship between higher education investments and the urban-rural income gap across China’s eight major comprehensive economic zones, constructing the following fixed effects model:


$${Gap}_{i,t}=\beta_{0}+\beta_{1}{Edu}_{i,t}+\beta_{2}{Controls}_{i,t}+\varphi_{i}+\varepsilon_{i,t}$$ (1)

In this model, the dependent variable Gap represents the urban-rural income disparity; the core explanatory variable Edu signifies higher education investment; control variables, denoted as Controls, represent the related control variables that can influence the urban-rural income gap; $\varphi_{i}$representing regional fixed effects; $\varepsilon_{i,t}$ represents the random error term.

Furthermore, technological innovation, serving as a catalyst for the development of the real economy, facilitates the harmonious development of society at large, thereby reducing the urban-rural divide. Moreover, due to the weaker economic development foundations in rural areas, technological innovation and its diffusion effects exhibit a more pronounced influence on enhancing farmers’ incomes [18,19]. Technological innovation cannot occur without high-tech talent; investment in higher education resources has, to a certain extent, increased the supply of high-quality human capital, providing talent support for technological innovation [20,21]. That is to say, investments in higher education may reduce the urban-rural income disparity through the intermediary mechanism of technological innovation (Ino).In view of this, the following mediation effect model is constructed on the basis of Equation (1):


$${Ino}_{i,t}=\nu_{0}+\nu_{1}{Edu}_{i,t}+\nu_{2}{Controls}_{i,t}+\varphi_{i}+\varepsilon_{i,t}$$ (2)

$${Gap}_{i,t}=\kappa_{0}+\kappa_{1}{Edu}_{i,t}+\kappa_{2}{Ino}_{i,t}+\kappa_{3}{Controls}_{i,t}+\varphi_{i}+\varepsilon_{i,t}$$ (3)

Additionally, the baseline panel model can demonstrate the impact of higher education investment on the urban-rural income gap, but it cannot prove the existence of a nonlinear relationship between the two. To avoid estimation errors caused by artificially setting threshold values and to accurately test the dividing points of the nonlinear relationship between higher education investment and the urban-rural income gap, this study employs Hansen’s (2000) panel threshold model to explore the relationship between higher education investment and the urban-rural income gap within different intervals of economic development levels [22]. Using the level of economic development (GDP) as the threshold variable, a single threshold model is established:


$${Gap}_{i,t}=\beta_{0}+\beta_{1}{Edu}_{i,t}(GDP\leq \gamma )+\beta_{2}{Edu}_{i,t}(GDP>\gamma )+\beta_{3}{Controls}_{i,t}+\varepsilon_{i,t}$$ (4)

where γ is the threshold value. If the results obtained from the single threshold model are significant and a single threshold exists, it is necessary to perform a double threshold model test, and so on. Therefore, the double threshold model is set as:


$${Gap}_{i,t}=\beta_{0}+\beta_{1}{Edu}_{i,t}\left(GDP\leq \gamma_{1}\right)+\beta_{2}{Edu}_{i,t}\left(\gamma_{1}<GDP\leq \gamma_{2}\right)+\beta_{3}{Edu}_{i,t}(GDP>\gamma_{2})+\beta_{4}{Controls}_{i,t}+\varepsilon_{i,t}$$ (5)

### Variable descriptions

Regarding the measurement methods for the dependent variable (urban-rural income gap), existing literature primarily employs the ratio of urban to rural household incomes, the difference in per capita disposable income between urban and rural residents, and the Theil index for assessment [18,23–25]. Considering the changes in the growth rate of urban and rural incomes, this paper refers to the method of Chen Binkai and Lin Yifu (2013) and uses the relative gap to reflect the urban-rural income gap (Gap), namely the ratio of per capita disposable income of urban residents to the per capita net income of rural residents (after 2013, the per capita disposable income of rural residents). The larger the value, the greater the urban-rural disparity. Additionally, in the robustness test, the Theil index is selected as the alternative variable for the dependent variable.

Regarding the measurement of the core explanatory variable (higher education investment), in a narrow sense, higher education investment can be measured by the funding for higher education, while a broad definition of higher education investment can include measures of human, material, and financial resources [26,27]. Considering the diversified characteristics of higher education investment, this paper refers to the method of Cai Wenbo and Chen Niannian (2022) and establishes three primary indicators of human, material, and financial investment and ten secondary indicators to construct the higher education investment index (Edu), as shown in Table 1.

**Table 1:** Higher education investment indicators.

Goal Item	Primary Indicator	Secondary Indicator
Higher education investment	Human resources investment	Number of full-time teachers in regular higher education institutions (persons)
		Number of research staff in regular higher education institutions (persons)
		Number of postgraduate enrollments in regular higher education (persons)
		Number of undergraduate enrollments in regular higher education (persons)
	Material resources investment	Number of regular higher education institutions (institutions)
		Land area occupied by regular higher education institutions (square meters)
		Book collections in libraries of regular higher education institutions (ten thousand volumes)
		Teaching and research equipment assets in regular higher education institutions (ten thousand yuan)
	Financial resources investment	Expenditures on educational affairs by regular higher education institutions (thousand yuan)
		Capital expenditure on basic construction by regular higher education institutions (thousand yuan)

Regarding the measurement of the mediating variable (level of technological innovation), it is commonly observed that the number of patent grants and patent applications are the most prevalent indicators reflecting the level of technological innovation [28].Considering the phenomenon of a “patent bubble” in the number of patent applications, this study, following the approach of scholar Shao Xingdong (2020), adopts the number of patent grants (Ino) to represent the level of technological innovation.

The impact of different stages of economic development on the urban-rural income gap varies [29]. Regarding the measurement of the threshold variable (level of economic development), existing literature primarily employs per capita GDP and real GDP as metrics to gauge the level of economic development [30,31].Considering that regional GDP can more accurately reflect a region’s economic scale and overall economic strength, this study selects the actual annual GDP of a region as the threshold variable to measure the local economic development condition.

To more accurately measure the impact of higher education investment on the urban-rural income gap in China’s eight major comprehensive economic zones, and to minimize the errors caused by other factors to the greatest extent. In existing research, factors that mainly affect the urban-rural income gap include economic development, urbanization, marketization, openness to foreign trade, financial development, and educational progress [29]. Given that a higher birth rate is associated with lower levels of urbanization and a larger urban-rural income gap, a greater degree of economic openness tends to narrow the urban-rural gap, financial efficiency has a negative relationship with the urban-rural income disparity, regions with a high proportion of agriculture in their GDP are economically backward and have a larger urban-rural income gap, and the consumption disparity between urban and rural residents leads to an income gap [32–36].Thus, the study selects six indicative control variables, specifically: Urbanization Level (Urban), measured by the proportion of the urban population to the total population at year-end; Birth Rate (Birth), measured by the proportion of births to the total population; Degree of Economic Openness (Open), measured by the ratio of the total volume of imports and exports to GDP, converted at the annual average exchange rate from USD to RMB; Financial Efficiency Level (Fin), measured by the ratio of deposits to loans in the region; Industrial Level (Industry), measured by the proportion of agriculture to GDP; and Urban-Rural Consumption Disparity (Consume), measured by the ratio of urban resident consumption expenditure to rural resident consumption expenditure.

### Data sources and descriptive statistical analysis

To ensure the accessibility and validity of the data, this study selects panel data from the eight major comprehensive economic zones in China for the years 2003–2021. On one hand, the eight major comprehensive economic zones, as delineated by the Development Research Center of the State Council to implement the regional coordinated development planning, are based on principles of geographical proximity, similarity in natural environments and factor structures, comparable levels of economic development, economic interconnections, or facing similar real-world challenges. These principles were applied to subdivide the four major regions of China—Northeast, East, West, and Central—into more detailed segments, facilitating the analysis of regions and homogenous units within regions. Specifically, these zones are divided into the Northern Coastal Comprehensive Economic Zone (Beijing, Tianjin, Hebei, Shandong), Eastern Coastal Comprehensive Economic Zone (Shanghai, Jiangsu, Zhejiang), Southern Coastal Comprehensive Economic Zone (Fujian, Guangdong, Hainan), Northeast Comprehensive Economic Zone (Liaoning, Jilin, Heilongjiang), Middle Yellow River Comprehensive Economic Zone (Shaanxi, Shanxi, Henan, Inner Mongolia), Middle Yangtze River Comprehensive Economic Zone (Hubei, Hunan, Jiangxi, Anhui), Greater Southwest Comprehensive Economic Zone (Yunnan, Guizhou, Sichuan, Chongqing, Guangxi), and Greater Northwest Comprehensive Economic Zone (Gansu, Qinghai, Ningxia, Tibet, Xinjiang). On the other hand, prior to 2003, statistical data on higher education investment indicators were relatively coarse, and 2003 marked the first year of a surge in graduates following the expansion of college admissions, a factor that could significantly impact the urban-rural income gap. Data for 2022 has not yet been published.

The dependent variable, core explanatory variable, threshold variable, and control variables in this study are all derived from the “China Statistical Yearbook,” “China Education Statistics Yearbook,” and “China Education Funds Statistical Yearbook.” Descriptive statistics for each variable are presented in Table 2. Examining the dependent variable, the urban-rural income ratio, from 2003 to 2021, the average per capita disposable income of urban residents in the eight major comprehensive economic zones of China was 2.759 times that of rural residents, with a minimum value of 1.842 times and a maximum value of 5.238 times. This indicates a significant and substantial income disparity between urban and rural areas within these economic zones. In terms of the core explanatory variable, higher education investment, the average was 0.247, with a minimum of 0.02 and a maximum of 0.656, suggesting that investment in higher education has been increasing but with relatively large discrepancies in the intensity of investment. The intermediary variable, the level of technological innovation, showed a relatively large gap between the minimum and maximum values, indicating a significant improvement in the technological innovation capability within the sample regions of the eight major comprehensive economic zones. For the threshold variable, the level of economic development, the average was 18647.11, with a minimum of 184.5 and a maximum of 124369.7, highlighting that economic development in these zones is improving but with large disparities in development levels. Looking at a series of control variables, such as the level of urbanization, population birth rate, and the urban-rural consumption gap, the differences between the minimum and maximum values are substantial, suggesting that the level of urbanization in these zones is relatively poor, population birth distribution is uneven, and there is a significant gap between rural and urban consumption levels. Furthermore, the gaps between the minimum and maximum values for openness, financial development efficiency, and industrial level are relatively small, indicating to some extent that the degree of openness and financial development efficiency in these economic zones may not have significantly increased during the selected period, and the proportion of agriculture in GDP has been relatively stable with a relatively small decrease.

**Table 2:** Descriptive statistics of variables.

Variable	Sample Size	Mean	Standard Deviation	Minimum Value	Maximum Value
Gap	589	2.759	.512	1.842	5.238
Edu	589	.247	.156	.002	.656
Ino	589	41773.23	85362.24	16	872209
GDP	589	18647.11	.19474.19	184.5	124369.7
Urban	589	53.306	15.112	20.11	89.6
Birth	589	11.051	2.936	3.59	17.94
Open	589	.301	.374	.008	1.843
Fin	589	1.362	.362	.839	4.3
Industry	589	.103	.058	.003	.436
Consume	589	2.717	.783	1.5	7.2

## Results

### Baseline regression model results and analysis

Fixed-effects regression was conducted based on the model presented in Equation (1), with the results displayed in Table 3. In Model 1, without the inclusion of control variables, the regression coefficient for Edu is negative and significant at the 1% statistical level, indicating that overall higher education investment in China’s eight major comprehensive economic regions can promote the narrowing of the urban-rural income gap. Further examination reveals that in Model 2, even after controlling for variables such as urbanization level, birth rate, degree of economic openness, financial efficiency development, industrial level, and the urban-rural consumption gap, the coefficient estimate remains significantly negative. In terms of effect size, a 1% increase in higher education investment in the Northeast region is associated with a 0.209 reduction in the urban-rural income disparity.

**Table 3:** Baseline regression results.

Variable	Model 1	Model 2
Edu	−1.901^***^ (.109)	−.209^**^ (.090)
Urban		−.008 ^***^ (.002)
Birth		.006 (.004)
Open		−.069 (.423)
Fin		.110^***^ (.024)
Industry		.425^*^ (.225)
Consume		.260^***^ (.014)
_cons	3.229^***^ (.029)	2.291^***^ (.138)
R-sq	.446	.687
Provincial fixed effects	Controlled	Controlled
N	589	589

Values in parentheses are standard errors. ***, **, and * indicate significance of the coefficients at the 1%, 5%, and 10% significance levels, respectively. The same applies below.

**Fig 2:**
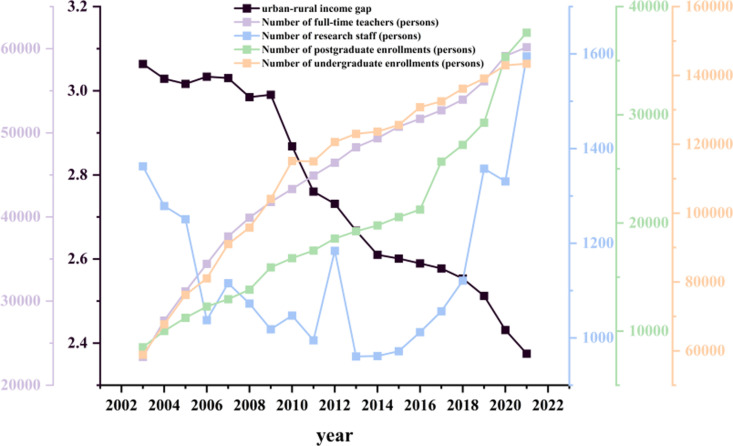
Average human capital investment in higher education and urban-rural income gap in China’s eight major comprehensive economic regions (2003-2021).

The underlying reason can be traced to trends in human capital investment in higher education. As shown in Fig 2, from 2003 to 2021, higher education institutions across China’s eight major comprehensive economic regions experienced steady growth in human resource inputs. The average numbers of full-time faculty, postgraduate enrollments, and undergraduate enrollments all increased, with the growth in postgraduate enrollment being particularly significant. This indicates the continuous expansion in both scale and academic level within the higher education system. Notably, the sustained expansion of higher education has markedly improved access for rural students. In recent years, the Ministry of Education has issued a series of policy directives mandating key universities to increase their enrollment proportions for students from rural and impoverished areas, thereby promoting the reallocation of educational resources toward rural regions. Relevant studies have found that, compared to their urban counterparts, rural household-registered students who attend top-tier universities achieve higher returns on education. Furthermore, entrepreneurship education at these institutions has effectively encouraged rural graduates to return to their hometowns to start businesses, thereby broadening income channels for rural residents and enhancing wage income levels [37–39].

In addition, the rapid expansion of higher education has continuously enhanced the overall stock of human capital in society, reduced the scarcity of highly educated individuals, alleviated the structural imbalance between the supply and demand of educational resources, and promoted the rational flow and optimal allocation of talent between urban and rural areas. These dynamics have exerted a compressive effect on the urban-rural income gap, contributing to its gradual convergence [40,41]. Moreover, the increasing number of university research personnel has facilitated agricultural technological innovation and the transformation of scientific achievements, thereby improving agricultural productivity and optimizing the structure of rural industries [42]. Notably, the development of emerging sectors such as digital agriculture, e-commerce, and rural service industries is closely linked to the technical and human resource support provided by the higher education system. This, in turn, has effectively fed back into the rural economy and strengthened income growth momentum in rural areas.

**Fig 3:**
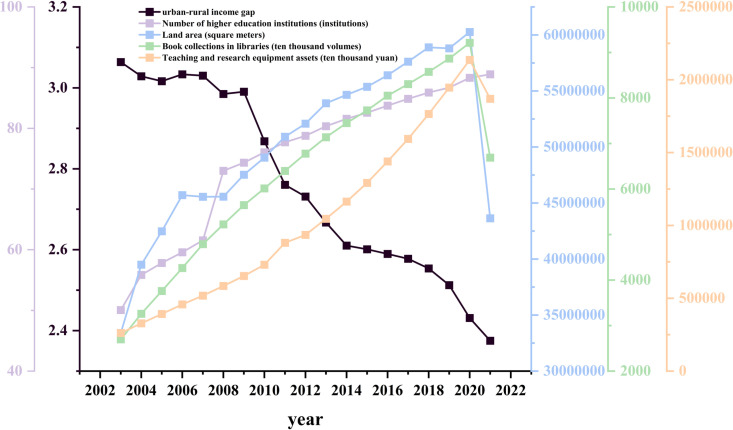
Average material investment in higher education and urban-rural income gap in China’s eight major comprehensive economic regions (2003-2021).

From the perspective of material investment in higher education, Fig 3 shows that from 2003 to 2021, the number of higher education institutions in China’s eight major comprehensive economic regions increased steadily, with growth stabilizing after 2008. This reflects the continuous expansion of higher education institutions and the sustained enhancement of their service capacity. Relevant studies have shown that the density of higher education institutions significantly promotes enterprise innovation, which in turn increases non-agricultural employment opportunities and facilitates income growth for rural residents, thereby helping to alleviate the urban-rural income gap to a certain extent [43]. In addition, indicators such as campus area, library holdings, and the value of teaching and research equipment assets maintained rapid growth between 2003 and 2020, substantially strengthening the material foundation of higher education. However, all three indicators experienced varying degrees of decline in 2021. Although this fluctuation did not alter the long-term trend of higher education resource expansion, it may be attributed to the sustained impact of major public crises during that year.Studies have further indicated that the enhancement of “new quality productive forces” contributes to narrowing the urban-rural income gap, and within the synergy between human capital and physical capital, human capital plays a more prominent role in driving the development of such productive forces [44,45]. Therefore, although higher education’s material investment declined temporarily in 2021, its impact on the urban-rural income gap remains limited from a long-term development perspective. Given the continued growth in human resource investment in higher education, the system retains its capacity to effectively promote the narrowing of urban-rural income disparities.

**Fig 4:**
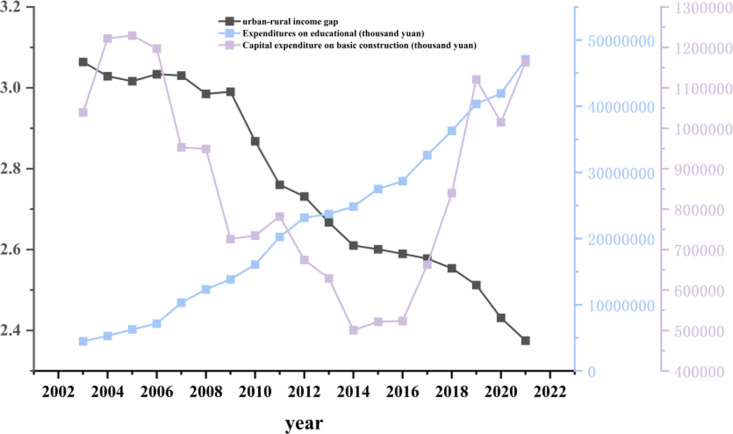
Average financial investment in higher education and urban-rural income gap in China’s eight major comprehensive economic regions (2003-2021).

From the perspective of financial investment in higher education, Fig 4 shows that from 2003 to 2021, expenditures on educational operations in higher education institutions across China’s eight major comprehensive economic regions exhibited a sustained and stable growth trend. In contrast, capital expenditures on basic construction followed a distinct U-shaped pattern—rising during the early years, declining mid-period, and rebounding in the later years.Generally, the continuous increase in operational expenditures has enabled universities to deepen investment in areas such as faculty development, talent cultivation, and research activities, thereby promoting both the expansion and qualitative improvement of human capital. Relevant studies indicate that basic construction expenditures are primarily focused on the expansion of physical resources, and their marginal contribution to enhancing universities’ innovation capacity is relatively lower than that of operational expenditures [46].The innovative talents trained by universities, along with the transformation of scientific and technological achievements, can effectively serve rural areas by facilitating their integration into the modern economic system. This process supports rural industrial upgrading and income diversification, thereby promoting farmer income growth and narrowing the urban-rural income gap [47].

To enhance the reliability of the research conclusions, the following three methods were employed for robustness checks: Firstly, an endogeneity test was conducted. Generally, the impact of the urban-rural income gap on higher education investment in China’s eight major comprehensive economic regions is minimal. Nonetheless, it is undeniable that omitted variables or measurement errors related to higher education investment may lead to econometric biases, resulting in endogeneity issues. This paper further employs the instrumental variable method to mitigate these endogeneity concerns. Drawing from Ma Lei (2017), the number of regular high schools in a region is selected as the instrumental variable, which satisfies the relevance and exogeneity conditions: on one hand, the more regular high schools in a region, the higher the proportion of local students, indicating greater local investment in higher education; on the other hand, considering that the quantity of high schools is determined by national educational authorities based on educational development needs, it is unlikely to affect the urban-rural income disparity within the region [48]. Column 1 of Table 4 shows the regression results of the instrumental variable, indicating that even after considering potential endogeneity between higher education investment and the urban-rural income gap, the coefficient of higher education investment remains negative. This suggests that higher education investment can narrow the urban-rural income gap, consistent with previous results. Moreover, this study addresses the issue of a weak instrumental variable; the first-stage F-statistic is 123.6, which is above the empirical threshold of 10, confirming the strength of the chosen instrumental variable.

**Table 4:** Robustness tests.

Variable	Test for endogeneity	Substitution of the dependent variable	Substitution of the core explanatory variable
Edu	−.954^***^ (.194)	−.105^**^ (.050)	−.057^***^ (.022)
Urban	−.005^***^ (.002)	−.000 (.001)	−.007^***^ (.002)
Birth	.011^***^ (.005)	−.004^*^ (.002)	.006 (.004)
Open	−.048 (.041)	.026 (.024)	−.056 (.042)
Fin	.066^*^ (.036)	.003 (.013)	.097^***^ (.024)
Industry	−.191 (.239)	.048 (.124)	.346 (.224)
Consume	.313^***^ (.029)	.031^***^ (.008)	.260^***^ (.014)
_cons	2.249 (.216)	.091 (.076)	2.814^***^ (.235)
R-sq	.722	.466	.678
Provincial fixed effects	Controlled	Controlled	Controlled
N	589	589	589

Secondly, substituting the dependent variable.Considering that the urban-rural income gap in our country primarily reflects changes at both ends of the spectrum, and the Theil index exhibits stronger sensitivity to income changes in both high-income and low-income groups, the Theil index is selected as an alternative measure of the urban-rural income gap [49]. The results in Column 2 of Table 4 show that its coefficient and impact are essentially consistent with those obtained when the urban-rural income gap is regressed as the dependent variable using the relative difference.

Thirdly, substituting the core explanatory variable. The sources of higher education investment primarily rely on government fiscal support [50]. Following the metrics chosen by Cai Wenbo and Huang Jinsheng (2016), and considering that per capita educational funding can reflect the extent of a region’s investment in higher education per student, the paper uses the expenditure on per capita educational funding in regular institutions of higher education as an alternative variable for higher education investment [51]. It is evident that the sign and significance of the estimated coefficients in Column 3 of Table 4 are fundamentally in agreement with the results of the regression using the higher education investment index derived from the entropy weight method as the core independent variable.

### Heterogeneous analysis on the sample of China’s eight major comprehensive economic zones

This study will further investigate the differential effects of the aforementioned influences across various regions, with the results presented in Table 5. Columns (1) through (8) represent the Northern Coastal Comprehensive Economic Zone, Eastern Coastal Comprehensive Economic Zone, Southern Coastal Comprehensive Economic Zone, Northeast Comprehensive Economic Zone, Middle Yellow River Comprehensive Economic Zone, Middle Yangtze River Comprehensive Economic Zone, Greater Southwest Comprehensive Economic Zone, and Greater Northwest Comprehensive Economic Zone, respectively.

**Table 5:** Results of heterogeneity analysis.

Variable	(1)	(2)	(3)	(4)	(5)	(6)	(7)	(8)
Edu	−.534^***^ (.144)	−1.162^***^ (.084)	.192 (.148)	−.894^***^ (.144)	.511^**^ (.212)	−.703^***^ (.243)	.613 (.563)	.417 (.547)
Urban	.002 (.002)	.001 (.002)	−.011^***^ (.002)	−.004 (.005)	−.023^***^ (.004)	−.017^***^ (.003)	−.034^***^ (.005)	−.004 (.005)
Birth	.006 (.005)	.015^***^ (.004)	.018^***^ (.005)	.005 (.017)	−.007 (.014)	.003 (.007)	.023^*^ (.014)	.015 (.014)
Open	.093^**^ (.040)	−.019 (.035)	.167^***^ (.049)	−.687^**^ (.290)	−2.228^***^ (.471)	1.124^**^ (.453)	−.568 (.389)	−1.347^***^ (.442)
Fin	.278^***^ (.064)	.077 (.061)	.150^***^ (.056)	.201^**^ (.076)	.076 (.084)	−.126 (.087)	.175 (.120)	.158^***^ (.037)
Industry	.065 (.546)	−1.182^*^ (.708)	.309 (.294)	−.018 (.235)	−.406 (.697)	.485 (.420)	.897 (.665)	.385 (.819)
Consume	.165^***^ (.029)	.098^***^ (.034)	.121^***^ (.018)	.140^***^ (.050)	.130^***^ (.041)	.033^*^ (.019)	.083^**^ (.037)	.406^***^ (.032)
_cons	1.526^***^ (.254)	2.241^***^ (.314)	2.335^***^ (.215)	2.429^***^ (.490)	3.754^***^ (.396)	3.483^***^ (.190)	3.762^***^ (.382)	1.725^***^ (.402)
R-sq	.855	.923	.922	.923	.879	.926	.895	.876
Provincial fixed effects	Controlled	Controlled	Controlled	Controlled	Controlled	Controlled	Controlled	Controlled
N	76	57	57	57	76	76	95	95

It is evident that higher education investment in 50% of China’s comprehensive economic zones can narrow the urban-rural income gap. These zones include the Northern Coastal Comprehensive Economic Zone, Eastern Coastal Comprehensive Economic Zone, Northeast Comprehensive Economic Zone, and the Middle Yangtze River Comprehensive Economic Zone. Among them, the Eastern Coastal Comprehensive Economic Zone exhibits the strongest convergence effect of higher education investment on the urban-rural income gap, with an effect coefficient of 1.162 for every 1% increase in higher education investment. The reason behind this is that the Eastern Coastal Comprehensive Economic Zone is one of the most influential multifunctional manufacturing centers, attracting a significant amount of domestic and foreign investment, which in turn increases local employment opportunities. Moreover, this region boasts rich higher education resources, including many of China’s top universities and research institutions. The “trickle-down” effect of higher education investment provides more educational opportunities for rural students in the region. Furthermore, the labor market has an urgent demand for talents with higher education, enhancing the chances for rural residents to migrate to cities for employment and, thereby, narrowing the urban-rural income gap [52].

Ranked second is the Northeast Comprehensive Economic Zone. Since 2003, China has been implementing the Northeast Revitalization Strategy. The “Several Opinions of the CPC Central Committee and the State Council on Fully Revitalizing the Old Industrial Bases in the Northeast Region,” issued in 2016, proposed the promotion of close integration between science and education institutions and regional development, supporting the accelerated development of research institutes, universities, and vocational colleges in the Northeast. The goal is to leverage the higher education levels in the Northeast to assist in the social development of the Northeast Comprehensive Economic Zone. Moreover, in September 2023, General Secretary Xi Jinping presided over a symposium on promoting comprehensive revitalization of the Northeast in the new era and emphasized in a significant speech, “It is necessary to improve the overall quality of the population to support the comprehensive revitalization of the Northeast with high-quality population development [53].” This illustrates that a series of supportive policies for the Northeast Comprehensive Economic Zone have enabled investments in higher education to somewhat narrow the urban-rural income gap.

Ranked third is the Middle Yangtze River Comprehensive Economic Zone. This region, spanning east to west, is the geographic center of the nation, characterized by strong economic driving forces and significant potential, with marked improvements in development quality and efficiency. However, the primary issues hindering the urban and rural development of the Middle Yangtze River Comprehensive Economic Zone are the shortcomings in education levels and innovation capacity. Therefore, the enhancement of human capital and innovation capabilities resulting from increased investments in higher education can propel the integrated development of urban and rural areas, aiming to narrow the urban-rural income gap [54].

Ranked fourth is the Northern Coastal Comprehensive Economic Zone. This zone has reached the highest level of educational resources in the country. However, the relative scale and level of higher education in Hebei Province are somewhat limited, restricting to some extent the Northern Coastal Comprehensive Economic Zone and leading to uneven development of higher education within the region [55]. Therefore, there remains some potential for investments in higher education to reduce the urban-rural income gap in the Northern Coastal Comprehensive Economic Zone.

It is worth mentioning that, in the Middle Yellow River Comprehensive Economic Zone, investment in higher education has widened the urban-rural income gap at a 5% significance level. In contrast, investments in higher education in the Southern Coastal Comprehensive Economic Zone, the Great Southwest Comprehensive Economic Zone, and the Great Northwest Comprehensive Economic Zone all have a positive effect on the urban-rural income gap, but they do not pass the significance test. The reason behind this, within the sample study period, is that the “competition effect” of increased labor quantity due to higher education investment in the Middle Yellow River Comprehensive Economic Zone did not significantly reduce the return rate of higher education. In other words, although higher education investment increased the overall level of education in the short term, the lack of supporting policies to promote equal educational opportunities led to an exacerbation of urban-rural income inequality in the Middle Yellow River Comprehensive Economic Zone. For the provinces within the Southern Coastal Comprehensive Economic Zone, Guangdong Province, as the most economically vibrant province, experiences a spatial spillover effect from increased investment in higher education, which can intensify the degree of urban-rural income inequality in neighboring provinces [17]. For the Great Southwest and Great Northwest Comprehensive Economic Zones, most provinces are relatively economically backward with limited capacity to develop education. Coupled with urban-biased education policies, educational resources in economically underdeveloped rural areas are further diminished, leading to minimal effects of income increase through education. This imbalance in higher education further causes inequalities in employment opportunities, positions, and compensations between urban and rural residents, thereby widening the income gap [12].

## Further analysis: Mediation mechanisms and threshold effects

### Analysis of mediation mechanism

The baseline regression results indicate that, overall, higher education investment in China’s eight major comprehensive economic zones can indeed narrow the urban-rural income gap. To explore how higher education investment in these zones contributes to narrowing this gap, this study discusses the issue from the perspective of facilitating technological innovation development. Table 6 presents the mediation effect regression results on how higher education investment in China’s eight major comprehensive economic zones affects the urban-rural income gap through technological innovation. Model 1 shows the primary effect regression results, with the coefficient of the core explanatory variable being significantly negative, indicating that higher education investment in the eight major comprehensive economic zones can reduce the urban-rural income disparity. Model 2 verifies whether higher education investment in these zones has an impact on the level of technological innovation, with the coefficient for higher education investment being 0.905 and significantly positive. Model 3 examines whether the level of technological innovation acts as a mediator between higher education investment in the northeastern region and the urban-rural income gap, with the technological innovation level coefficient being −0.191 and significant, indicating the presence of a mediation effect. The significant regression coefficient of higher education investment demonstrates that the level of technological innovation partially mediates the relationship between higher education investment and the urban-rural income disparity.

**Table 6:** Results of the mediation effect test.

Variable	Model 1 (Gap)	Model 2 (Ino)	Model 3 (Gap)
Edu	−.209^**^ (.090)	.905^***^ (.157)	−.036^***^ (.023)
Ino			−.191^***^ (.023)
Controls	Controlled	Controlled	Controlled
_cons	2.291^***^ (.138)	6.058^***^ (.240)	3.451^***^ (.191)
R-sq	.687	.892	.807
Provincial fixed effects	Controlled	Controlled	Controlled
N	589	589	589

The reasons for this can be attributed to two main factors. On one hand, education is a quasi-public good with positive externalities, primarily funded by government fiscal support in China. This arrangement can circumvent potential supply shortages that may arise from market provision. By investing in higher education, it is possible to accelerate the flow and diffusion of elements such as knowledge and technology, thereby fostering the formation of new ideas and concepts. This, in turn, has a positive impact on the region’s enthusiasm for and success rate of autonomous innovation [56]. On the other hand, global economic instability, exacerbated by the global pandemic, the Russo-Ukrainian conflict, and major power strategic competition, has precipitated profound transformations in the education system [57,58]. In fact, the digitalization of education has become a new imperative to adapt to economic and social development, address the contradiction between social development and the supply and demand of talent, and enhance social productivity [59]. Currently, increased investment in higher education can to some extent effectively advance the transformation of higher education online teaching. Simultaneously, the innovation and development needs of online education services are guiding the pace, depth, and direction of technological innovation. Furthermore, digital investment in higher education enables universities to leverage their distinctive disciplinary advantages, broaden research areas and main research directions, and aggregate high-quality resources. This facilitates the establishment of an autonomous innovation research system with complete intellectual property rights owned by the institution, thereby accelerating the transformation of autonomous innovation outcomes [60].

Furthermore, technological innovation development enhances the productivity of non-agricultural industries, promoting industrialization and urbanization. This, in turn, allows for industry to feed back into rural development, meaning rural areas can integrate with urban industrial chains to achieve industrial upgrading and economic structural optimization. This “town-leading-village” development model enables rural areas to share in the economic benefits of urbanization, thus driving integrated urban-rural development. Additionally, advancements in agricultural technology, such as precision agriculture, smart farming equipment, and biotechnology, have significantly increased agricultural productivity. These advancements also promote the diversification and value addition of agricultural products, expanding the development space of the rural economy, enhancing the competitiveness of the rural market, thereby increasing farmers’ incomes and narrowing the urban-rural income gap [61].It is noteworthy that, under the guidance of new development concepts, agricultural green development has become a theme of the era. Green resources have become key to rural areas in breaking the “development paradox” and forming a comparative advantage in rural development, increasingly considered as a new factor in promoting farmers’ income growth. In other words, green technological innovation and progress in agriculture can increase the maximum output achievable in agricultural production, thereby helping to improve farmers’ net income, household operational income, and wage income, ultimately aiming to narrow the urban-rural income gap [62,63].

Considering the inherent endogeneity issues within the mediation effect, this study employs the ivmediate model proposed by Dippel et al. (2020) for causal mediation analysis of the mediation effect model mentioned above [64]. By using the lagged level of technological innovation as an instrumental variable, this analysis tests for direct and indirect effects after addressing endogeneity concerns, as shown in Table 7. According to the regression results in Table 7, the total effect estimation indicates that for every one percentage point increase in higher education investment in China’s eight major comprehensive economic zones, the urban-rural income gap narrows by 1.09 percentage points. The direct effect estimation shows that only 0.490 percentage points of the urban-rural income gap reduction are attributable to higher education investment, significant at the 1% level. The indirect effect estimation reveals that 0.600 percentage points of the income gap reduction are caused by mediating factors. Thus, this confirms the mediating role of the level of technological innovation in the relationship between higher education investment and the urban-rural income gap in China’s eight major comprehensive economic zones.

**Table 7:** Results of the mediation effect test.

Causal mediation effect regression parameter	Causal mediation effect decomposition		
	Total effect	Direct effect	Indirect effect
Coefficient	−1.090	−.490	−.600
Std. err.	.146	.166	.242
z	−7.490	−2.950	−2.480
P > |z|	.000	.003	.013
[95% conf. interval]	[-1.379, -.807]	[-.816, -.165]	[-1.074,-.125]
N	558	558	558

### Analysis of threshold effects

This study initially conducts a comprehensive test to determine whether a threshold effect and the number of thresholds exist between higher education investment and the urban-rural income gap across China’s eight major comprehensive economic zones. Drawing on the Bootstrap method proposed by HANSEN (1999) to estimate the F-statistic, this method tests for both double and single thresholds by resampling 300 times using the Bootstrap method [65]. The results, as shown in Table 8, indicate that under a single threshold setting, the p-value corresponding to the F-statistic is 0.060, suggesting significance at the 10% level. This implies that the regression model exhibits a single threshold effect, with the threshold value identified at 5.835.

**Table 8:** Results of the threshold effect test.

Model	Threshold value	F-Statistic	P-Value	1% Critical value	5% Critical value	10% Critical value
Single threshold	5.835^*^	41.63	.060	36.205	42.265	64.181
Double threshold	10.296	32.99	.103	33.027	39.176	60.720

Further examination reveals that the fixed effects regression results of the threshold model are as shown in Table 9. Overall, these results indicate that, with the level of economic development as the threshold, there is a significant nonlinear relationship between higher education investment and the urban-rural income gap across China’s eight major comprehensive economic zones. Specifically, when lnGDP ≤ 5.835, the regression coefficient of higher education investment on the urban-rural income gap in these zones is 99.242, significant at the 1% statistical level. Conversely, when lnGDP > 5.835, the regression coefficient of higher education investment on the urban-rural income gap is −0.356, significant at the 10% statistical level.

**Table 9:** Threshold regression results.

Variable	Coefficient
Edu(lnGDP ≤ 5.835)	99.242^***^ (22.284)
Edu(lnGDP > 5.835)	−.356^*^ (.200)
Controls	Controlled
_cons	2.520^***^ (.555)
R-sq	.804
N	589

This indicates that the impact of higher education investment on the urban-rural income gap exhibits nonlinear characteristics across China’s eight major comprehensive economic zones, depending on the level of economic development. Upon examination, overall, the investment in higher education within China’s eight major comprehensive economic zones exhibits a “Kuznets effect”. At lower stages of economic development (lnGDP ≤ 5.835), investment in higher education may exacerbate the unequal distribution of resources, allowing urban areas to develop more rapidly due to the concentration of educational resources, thereby further widening the income gap with rural areas. However, at higher levels of economic development (lnGDP ＞ 5.835), increased investment in higher education is more often utilized to support research and development, innovation, and the cultivation of high-level talents. These activities contribute to technological advancement and industrial upgrading. In other words, the optimization of human capital structure and technological progress brought about by higher education investment can promote the development of the secondary and tertiary industries. The development of the secondary industry can support agriculture, thus promoting agricultural modernization and raising the income of residents in the agricultural sector. The development of the tertiary industry, by absorbing surplus rural labor, can enhance the income levels of this labor force, ultimately achieving the goal of narrowing the urban-rural income gap [66]. Moreover, under the synergistic effects of the rural revitalization strategy and innovation and entrepreneurship policies, increased investment in higher education is conducive to encouraging rural university graduates to return to their hometowns for entrepreneurship. The numerous jobs created by entrepreneurship can absorb the surplus labor force in rural areas, thereby promoting entrepreneurship and employment through multiple channels, achieving the purpose of narrowing the urban-rural income gap [67].

Similarly, a specific threshold regression analysis was conducted for China’s eight major comprehensive economic zones, with the results of the threshold value tests shown in Table 10. Columns (1) – (8) represent the Northern Coastal Comprehensive Economic Zone, Eastern Coastal Comprehensive Economic Zone, Southern Coastal Comprehensive Economic Zone, Northeast Comprehensive Economic Zone, Middle Yellow River Comprehensive Economic Zone, Middle Yangtze River Comprehensive Economic Zone, Greater Southwest Comprehensive Economic Zone, and Greater Northwest Comprehensive Economic Zone, respectively. From the threshold value test results in Table 10, it is known that the Eastern Coastal Comprehensive Economic Zone, Southern Coastal Comprehensive Economic Zone, Middle Yellow River Comprehensive Economic Zone, and Greater Southwest Comprehensive Economic Zone each passed the threshold value test at different significance levels. Among these, the Southern Coastal Comprehensive Economic Zone passed two threshold value tests, while the other three zones passed one threshold value test each. However, the Northern Coastal Comprehensive Economic Zone, Northeast Comprehensive Economic Zone, Middle Yangtze River Comprehensive Economic Zone, and Greater Northwest Comprehensive Economic Zone did not pass the threshold value test, meaning that these four regions do not have a threshold value.

**Table 10:** Results of the reginal threshold effect test.

Threshold	Test	(1)	(2)	(3)	(4)	(5)	(6)	(7)	(8)
Single	F-Statistic	4.580	18.670	9.590	6.010	29.530	10.500	23.780	11.920
	P-Value	.517	.100	.083	.440	.040	.377	.073	.347
	Threshold value	10.919	8.741^*^	8.790^*^	7.833	9.433^**^	9.355	8.464^*^	7.437
Double	F-Statistic			8.46					
	P-Value			.100					
	Threshold value			10.796^*^					

The threshold regression equations for the Eastern Coastal Comprehensive Economic Zone, Southern Coastal Comprehensive Economic Zone, Middle Yellow River Comprehensive Economic Zone, and the Greater Southwest Comprehensive Economic Zone are shown in columns (1) – (4) of Table 11. From the analysis results of the Eastern Coastal Comprehensive Economic Zone, it can be observed that when lnGDP ≤ 8.741, higher education investment reduced the urban-rural income gap, with an effect coefficient of −1.359. When lnGDP > 8.741, higher education investment also reduced the urban-rural income gap, but the negative effect was diminished, with an effect coefficient of −1.246. In other words, the convergence effect of higher education investment on the urban-rural income gap shows a decreasing effect. The reason behind this is that, compared to other comprehensive economic zones, the Eastern Coastal Comprehensive Economic Zone has a higher level of economic development within the sample range. At lower levels of economic development (lnGDP ≤ 8.741), the economy grows rapidly and there is a strong demand for talent, which also helps more rural residents to obtain opportunities to work in cities, thereby significantly narrowing the urban-rural income gap. At higher levels of economic development (lnGDP > 8.741), the overall economic size is large and resources for higher education are relatively abundant, resulting in smaller differences in opportunities for rural and urban residents to receive higher education. In this context, further increasing investment in higher education shows a diminishing marginal effect on narrowing the urban-rural gap.

**Table 11:** Regional threshold regression results.

Variable	(1)	(2)	(3)	(4)
	Coefficient	Coefficient	Coefficient	Coefficient
Edu(lnGDP ≤ 8.741)	−1.359^**^			
Edu(lnGDP > 8.741)	−1.246^**^			
Edu(lnGDP ≤ 8.790)		−.300^*^		
Edu(8.790 < lnGDP ≤ 10.796)		.291^**^		
Edu(lnGDP > 10.796)		−.303^*^		
Edu(lnGDP ≤ 9.433)			1.353^*^	
Edu(lnGDP > 9.433)			.573	
Edu(lnGDP ≤ 8.464)				2.519^*^
Edu(lnGDP > 8.464)				.333
Controls	Controlled	Controlled	Controlled	Controlled
_cons	2.391^**^	2.929^***^	3.137^***^	3.723^***^
R-sq	.928	.949	.937	.920
N	57	57	76	95

The threshold regression equations for the Eastern Coastal Comprehensive Economic Zone, Southern Coastal Comprehensive Economic Zone, Middle Yellow River Comprehensive Economic Zone, and the Greater Southwest Comprehensive Economic Zone are presented in columns (1) – (4) of Table 11. The analysis of the Eastern Coastal Comprehensive Economic Zone reveals that when lnGDP ≤ 8.741, investment in higher education reduces the urban-rural income gap, with an effect coefficient of −1.359. When lnGDP > 8.741, investment in higher education continues to reduce the urban-rural income gap, but the negative effect is diminished, with an effect coefficient of −1.246. This indicates that the role of higher education investment in narrowing the urban-rural income gap exhibits a decreasing effect. The rationale for this is that compared to other comprehensive economic zones, the Eastern Coastal Comprehensive Economic Zone experiences a higher level of economic development within the sample interval. At lower levels of economic development (lnGDP ≤ 8.741), the economy grows rapidly and there is a high demand for talent, which also helps more rural residents gain opportunities for urban employment, thereby significantly narrowing the urban-rural income gap. However, at higher levels of economic development (lnGDP > 8.741), the overall economic size is large, and resources for higher education are relatively abundant, making the differences in opportunities for higher education between rural and urban residents comparatively small. Under such circumstances, further increasing the investment in higher education shows a diminishing marginal effect on narrowing the urban-rural divide.

In the Southern Coastal Comprehensive Economic Zone, it is observed that when lnGDP ≤ 8.790, higher education investment decreases the urban-rural income gap, with an effect coefficient of −0.300. When lnGDP is between 8.790 and 10.796, higher education investment exacerbates the urban-rural income gap, with an effect coefficient of 0.291. However, when lnGDP > 10.796, higher education investment again reduces the urban-rural income gap, with an effect coefficient of −0.303. The reason for this pattern is that at moderate levels of economic development (lnGDP ≤ 8.790), the city-biased knowledge spillover effect brought about by higher education investment has a lag. Consequently, higher education investment can promote economic growth in rural areas to some extent, thereby reducing the urban-rural income gap. When the economic development level is at a medium stage (8.790 ＜ lnGDP ≤ 10.796), the regional economy grows rapidly, and higher education investment causes a more pronounced siphoning effect from urban to rural areas, exacerbating the income gap between urban and rural areas as the increasingly vulnerable rural agriculture deepens. In the case of Guangdong Province, compared to the relative dispersion of major cities in other provinces, economically developed cities in Guangdong are mostly concentrated in the Pearl River Delta. This undoubtedly increases the spatial distance between rural areas and developed cities, making it difficult for the spillover effects of economically developed cities to cover rural areas [68]. When the level of economic development is high (lnGDP > 10.796), the overall development momentum within the region is relatively coordinated. The knowledge dissemination and technological innovation brought about by higher education investment help enhance productivity in rural areas, narrowing the gap with urban areas.

In the Middle Yellow River Comprehensive Economic Zone, when lnGDP ≤ 9.433, higher education investments have exacerbated the urban-rural income gap, with an effect coefficient of 1.353. However, when lnGDP > 9.433, the impact of higher education investments on the change in the urban-rural income gap is not significant. Similarly, in the Greater Southwest Comprehensive Economic Zone, when lnGDP ≤ 8.464, higher education investments have intensified the urban-rural income disparity. Yet, when lnGDP > 8.464, the influence of higher education investments on the urban-rural income gap becomes insignificant. The underlying reasons for these phenomena in both the Middle Yellow River and Greater Southwest Comprehensive Economic Zones include geographical location, topography, ecological environment, and economic foundation. Increased investments in higher education attract a large rural population to cities for work, leading to a shortage and poor quality of human capital in rural areas. For instance, in Shanxi Province, mountainous areas account for over 80% of the region, and frequent natural disasters make agricultural development challenging. This scenario highlights a significant urban siphon effect of higher education investments, thereby exacerbating the urban-rural income disparity [69].

## Conclusion

This study utilizes panel data from China’s eight major comprehensive economic zones from 2003 to 2021 to analyze the impact of higher education investment on the urban-rural income gap within these zones and its transmission mechanism. Furthermore, it examines the nonlinear impact of higher education investment on the urban-rural income gap across different levels of economic development. The findings reveal, firstly, that overall, higher education investment significantly negatively affects the urban-rural income gap, meaning that increasing higher education investment in China’s eight major comprehensive economic zones can significantly reduce this gap. This conclusion holds true even after conducting a series of robustness tests, including the introduction of instrumental variables, replacement of dependent variables, and core explanatory variables. Secondly, specifically, higher education investment in 50% of these economic zones can narrow the urban-rural income gap, namely in the Northern Coastal Economic Zone, Eastern Coastal Economic Zone, Northeastern Economic Zone, and the Middle Yangtze River Economic Zone. However, higher education investment widened the income gap in the Middle Yellow River Economic Zone, Southern Coastal Economic Zone, the Greater Southwest Economic Zone, and the Greater Northwest Economic Zone, with only the Middle Yellow River Economic Zone passing the significance test. This indicates a distinct regional heterogeneity in the impact of higher education investment on the urban-rural income gap. Thirdly, mediation effect testing revealed that the level of technological innovation is the fundamental pathway through which higher education investment in China’s eight major comprehensive economic zones narrows the urban-rural income gap. This conclusion remains valid after robustness testing with instrumental variables. Fourthly, further research finds that overall, the impact of higher education investment on the urban-rural income gap in China’s eight major comprehensive economic zones is also affected by the level of economic development, exhibiting an “inverted U-shaped” characteristic with a single threshold value. Notably, this nonlinear impact varies regionally, with only the Eastern Coastal Economic Zone, Southern Coastal Economic Zone, Middle Yellow River Economic Zone, and the Greater Southwest Economic Zone passing the threshold value test. Specifically, the Southern Coastal Economic Zone passed two threshold value tests, while the others passed one, indicating a difference in this nonlinear relationship from the overall “inverted U-shaped” characteristic.

Based on the aforementioned conclusions, to better strengthen the role of higher education investment in narrowing the urban-rural income gap, this paper proposes the following policy recommendations:

First, given that higher education investment has been shown to effectively reduce the urban-rural income gap across China’s eight major comprehensive economic zones, it is essential to comprehensively advance overall investment in higher education within these regions. This includes promoting the deep integration of human, material, and financial resources, strengthening the capacity of higher education to serve agriculture, rural areas, and farmers (“San Nong”), and enhancing its supporting role in narrowing the urban-rural income gap.

Second, given the heterogeneity in the impact of higher education investment on the urban-rural income gap across different economic regions, it is necessary to optimize the investment structure based on local conditions and enhance the inclusiveness and coordinated development capacity of each region. For the Northern Coastal, Eastern Coastal, Northeast, and Middle Yangtze River Comprehensive Economic Zones, continued investment should be encouraged to fully leverage the spillover and demonstration effects of higher education investment in narrowing the urban-rural income gap.

Third, given the mediating role of technological innovation in the relationship between higher education investment and the urban-rural income gap, higher education should be leveraged to empower technological advancement. This can be achieved by strengthening the construction of university research platforms, promoting the transformation of scientific and technological achievements, and enhancing talent cultivation. Furthermore, innovation resources should be directed toward agriculture and rural industries to facilitate industrial upgrading and income growth, thereby improving the indirect effectiveness of higher education in narrowing the urban-rural income gap.

Fourth, considering that the impact of higher education investment on the urban-rural income gap is moderated by the level of economic development—and that this moderating effect varies across regions—it is essential to account for regional heterogeneity. Policymakers should align higher education investment strategies with the specific economic development stages of each region, scientifically identifying and managing the “critical threshold” of investment. Doing so can help prevent imbalances in investment structure that may otherwise exacerbate the urban-rural income gap at certain stages, thereby enhancing the precision and effectiveness of higher education in adjusting the urban-rural income disparity across different development phases.
